# Severe Thrombocytopenia after Zika Virus Infection, Guadeloupe, 2016

**DOI:** 10.3201/eid2304.161967

**Published:** 2017-04

**Authors:** Timothée Boyer Chammard, Kinda Schepers, Sébastien Breurec, Thierry Messiaen, Anne-Laure Destrem, Matthieu Mahevas, Adrien Soulillou, Ludovic Janaud, Elodie Curlier, Cécile Herrmann-Storck, Bruno Hoen

**Affiliations:** Centre Hospitalier Universitaire, Pointe-à-Pitre, France (T. Boyer Chammard, K. Schepers, S. Breurec, T. Messiaen, A.-L. Destrem, A. Soulillou, L. Janaud, E. Curlier, C. Herrmann-Storck, B. Hoen);; Institut National de la Santé et de la Recherche Médicale, Pointe-à-Pitre (K. Schepers, B. Hoen); Institut Pasteur de Guadeloupe, Pointe-à-Pitre (S. Breurec);; Université des Antilles, Pointe-à-Pitre (S. Breurec, B. Hoen);; Centre Hospitalier Universitaire Henri Mondor, Créteil, France (M. Mahevas)

**Keywords:** Zika virus, viruses, Zika, infection, thrombocytopenia, hemorrhagic signs and symptoms, Guadeloupe

## Abstract

Severe thrombocytopenia during or after the course of Zika virus infection has been rarely reported. We report 7 cases of severe thrombocytopenia and hemorrhagic signs and symptoms in Guadeloupe after infection with this virus. Clinical course and laboratory findings strongly suggest a causal link between Zika virus infection and immune-mediated thrombocytopenia.

Zika virus is an arthropod-borne flavivirus transmitted by mosquitoes. The most common signs and symptoms of Zika virus infection include pruritic rash, headache, arthralgia, myalgia, nonpurulent conjunctivitis, and low-grade fever (*1*). Signs and symptoms of Zika virus infection are usually mild. Rare complications, including birth defects, mainly microcephaly (*2*), and neurologic complications, such as Guillain-Barré syndrome, meningoencephalitis, and acute myelitis, might occur and have been reported during recent outbreaks (*3**–**6*). However, severe forms requiring hospitalization are uncommon, and lethality is low (*1*). In most cases, initial laboratory findings are nonspecific and whole blood cell counts are often within reference ranges (*7*). Mild-to-moderate thrombocytopenia has been rarely described (*8**,**9*), and severe thrombocytopenia has been reported only recently as an uncommon manifestation (*10**–**14*).

## The Study

We report severe thrombocytopenia (i.e., platelet count <50 × 10^9^/L) (*15*), which developed during or after the course of acute Zika virus infection in 7 patients who were admitted to the Guadeloupe University Hospital, French West Indies, during May–August 2016. This period coincides with the peak of a Zika outbreak in Guadeloupe.

The 7 patients (5 women and 2 men, mean age 43 years, range 15−74 years) had petechial purpura in the lower limbs (Table). Five of the patients also had additional bleeding signs and symptoms (gingival bleeding, epistaxis, oral hemorrhagic mucosal blisters, and hematuria). These manifestations prompted us to perform blood tests, which showed isolated thrombocytopenia. Results of physical examinations were otherwise unremarkable. All 7 patients had a typical Zika virus infection (median 5 days, range 2–18 days) before diagnosis of thrombocytopenia. Median minimum platelet count was 2 × 10^9^/L (range 1 × 10^9^/L–17 × 10^9^/L). Results of peripheral blood smears were unremarkable for all patients.

**Table:** Characteristics of 16 patients with severe thrombocytopenia associated with Zika virus infection*

Pt no./ age, y/sex	Country	Hemorrhagic signs and symptoms	Min platelet count, × 10^9^/L	Days to min platelet count†						Steroid therapy	IVIG	Outcome	Reference
					RT-PCR result								
					Zika virus			DENV					
					Urine	Plas		Urine	Plas				
1/38/F	Guadeloupe	Yes	17	3	+	–		–	–	Yes	No	Recovered	This study
2/58/F	Guadeloupe	Yes	3	6	+	+		–	–	Yes	Yes	Recovered	This study
3/15/F	Guadeloupe	Yes	2	19	+	+		–	–	Yes	No	Recovered	This study
4/36/M	Guadeloupe	Yes	5	8	+	–		–	–	Yes	No	Recovered	This study
5/74/F	Guadeloupe	Yes	1	5	+	–		–	–	Yes	No	Recovered	This study
6/46/F	Guadeloupe	Yes	1	12	+	Unk		–	Unk	Yes	Yes	Recovered	This study
7/35/M	Guadeloupe	Yes	1	4	+	–		–	–	Yes	Yes	Recovered	This study
8/54/F	Suriname	Yes	10	29	+	–		–	Unk	No	Yes	Recovered	(*10*)
9/2/F	Colombia	Yes	<14	Unk	Unk	+		Unk	–	Unk	Unk	Died	*(11*)
10/30/F	Colombia	Yes	<14	Unk	Unk	+		Unk	–	Unk	Unk	Died	(*11*)
11/72/F	Colombia	Yes	<14	Unk	Unk	+		Unk	–	Unk	Unk	Died	(*11*)
12/72/M	Puerto Rico	Yes	1	5	Unk	+		Unk	–	No	No	Died	(*13*)
13/38/M	Puerto Rico	Yes	2	7	–	–		Unk	–	Yes	Yes	Recovered	(*13*)
14/26/F	Martinique	Yes	2	8	+	–		Unk	–	Yes	No	Recovered	(*14*)
15/21/M	Martinique	Yes	3	7	+	–		Unk	–	Yes	No	Recovered	(*14*)
16/30/F	Colombia	No	9	4	+	–		Unk	Unk	No	No	Recovered	(*12*)

*DENV, dengue virus; IVIG, intravenous immune globulins; min, minimum; plas, plasma; pt, patient; unk, unknown. †From Zika onset.

We evaluated patients for a differential diagnosis of isolated severe thrombocytopenia. None had recently received a new medication or vaccination or had traveled to an area to which malaria is endemic. No underlying conditions, such as autoimmune or lymphoproliferative disorders, were known or identified for any patient. Four patients had nonsignificantly positive antinuclear antibody titers (1:80–1:160). None of these patients had signs or symptoms of connective tissue disease.

Serologic test results for HIV and hepatitis B virus were negative for all 7 patients. Two of 7 patients had positive serologic results for hepatitis C virus; these 2 patients had negative results for hepatitis C virus RNA in plasma. Serologic test results ruled out diagnoses of acute leptospirosis, cytomegalovirus infection, and Epstein-Barr virus infection for all 7 patients. Six of 7 patients showed negative results for parvovirus B19 infection (1 patient was not tested). We did not test patients for chikungunya virus because this virus had not been detected in Guadeloupe since January 2015.

We tested patients for infection with Zika virus and dengue virus (DENV) by using reverse transcription PCR (RT-PCR) for urine samples <6 days of onset of purpura. Results of RT-PCR were positive for Zika virus and negative for DENV for all patients. In addition, results of serum tests for DENV nonstructural protein 1 were negative for all patients. IgG against DENV was detected in 6 of the 7 patients.

A diagnosis of acute immune mediated thrombocytopenia (ITP) was made for all 7 patients. Because of thrombocytopenia severity, all patients received steroid therapy with either prednisone or methylprednisolone at an initial dosage of 1–2 mg/kg/day. Three patients also received intravenous immune globulins (IVIG). Two patients received platelet transfusions. Except for patient 2, platelet counts returned to reference ranges <15 days after treatment initiation for all patients. After >2 months without treatment, no relapse was observed in any patient. We provide additional information on the atypical clinical course that was observed for 3 of the patients.

Patient 1 had a history of refractory ITP. She had been treated for primary ITP during 2004–2007 with steroids and IVIG, followed by vinblastine and danazol, and eventually splenectomy, which was curative. In 2014, an acute chikungunya virus infection caused a relapse of ITP, which fully responded to a short-course steroid treatment. During 2014–2016, she remained asymptomatic and had a platelet count >100 × 10^9^/L. In May 2016, she was hospitalized 2 days after onset of a typical Zika virus infection. The patient had petechiae in the upper and lower limbs and a platelet count of 17 × 10^9^/L. Her clinical course rapidly became favorable after steroid therapy, and she had a platelet count of 172 × 10^9^/L by day 5 of steroid therapy. She was the only patient who did not have IgG against DENV.

Patient 2 responded only partially to steroids and IVIG and had a maximum platelet count of 92 × 10^9^/L at day 14 after treatment initiation. While she was underdoing tapering of steroid treatment, palate petechiae appeared on day 39 (platelet count 9 × 10^9^/L). Prednisone (1 mg/kg/day) was given for 10 days and was followed by a sustained recovery of the platelet count.

Patient 7 had severe hemorrhagic manifestations (gross hematuria and oral hemorrhagic blisters) and platelet count of 1 × 10^9^/L. He was hospitalized in an intensive care unit and received steroid therapy, IVIG, and platelet transfusion. The patient showed a full response to treatment (platelet count 169 × 10^9^/L at day 7 of treatment).

## Conclusions

From the beginning of the current Zika outbreak in the Americas to November 2016, nine case-patients with severe Zika virus–associated thrombocytopenia have been reported, 1 in Suriname (*10*), 4 in Colombia (*11**,**12*), 2 in Puerto Rico (*13*), and 2 in Martinique (*14*). We report information for these 9 case-patients and the 7 patients we analyzed in Guadeloupe (Table).

The 16 patients had similar characteristics. First, all had severe and profound thrombocytopenia (platelet counts <20 × 10^9^/L). Second, probably as a consequence of thrombocytopenia, hemorrhagic manifestations developed in all but 1 patient. Third, thrombocytopenia was present shortly after acute Zika virus infection, and Zika virus RNA was still detected in urine from 11 of the 12 patients for whom RT-PCR for Zika virus was performed.

Overall, despite the severity of thrombocytopenia, the outcome was generally favorable after conventional steroid treatment with or without IVIG. Among the 4 patients who died, only 1 patient had isolated thrombocytopenia; this patient died of hemorrhagic complications (*13*). The other 3 patients had various systemic signs and symptoms and thrombocytopenia; thrombocytopenia was the direct cause of death for only 1 patient (*11*).

For the 7 patients in Guadeloupe, we were able to exclude all other main causes of isolated severe thrombocytopenia, especially DENV infection. Because all 7 patients still had a positive RT-PCR result for Zika virus in urine when thrombocytopenia was diagnosed, we can reasonably assume that ITP was secondary to Zika virus infection. As reported for other viruses, Zika virus–associated ITP might result from stimulation of the immune system, which usually decreases after clearance of viral replication. The mechanism of thrombocytopenia was probably different in patients 1 and 16 (*12*), in whom thrombocytopenia appeared as a relapse of previous ITP.

In conclusion, thrombocytopenia is a rare complication of Zika virus infection. Our observations strongly suggest a causal relationship between Zika virus infection and ITP. Therefore, Zika virus should be included in the list of viruses that might trigger immune-mediated severe thrombocytopenia.

## References

[1] Brasil P, Calvet GA, Siqueira AM, Wakimoto M, de Sequeira PC, Nobre A, et al. Zika virus outbreak in Rio de Janeiro, Brazil: clinical characterization, epidemiological and virological aspects. PLoS Negl Trop Dis. 2016;10:e0004636. 10.1371/journal.pntd.0004636 27070912 PMC4829157

[2] de Araújo TV, Rodrigues LC, de Alencar Ximenes RA, de Barros Miranda-Filho D, Montarroyos UR, de Melo AP, et al.; investigators from the Microcephaly Epidemic Research Group; Brazilian Ministry of Health; Pan American Health Organization; Instituto de Medicina Integral Professor Fernando Figueira; State Health Department of Pernambuco. Association between Zika virus infection and microcephaly in Brazil, January to May 2016: preliminary report of a case-control study. Lancet Infect Dis. 2016 Sep 15;pii: S1473-3099(16)30318-8. 10.1016/S1473-3099(16)30318-8 PMC7617035 27641777

[3] Dos Santos T, Rodriguez A, Almiron M, Sanhueza A, Ramon P, de Oliveira WK, et al. Zika virus and the Guillain-Barré syndrome: case series from seven countries. N Engl J Med. 2016;375:1598–601. 10.1056/NEJMc1609015 27579558

[4] Carteaux G, Maquart M, Bedet A, Contou D, Brugières P, Fourati S, et al. Zika virus associated with meningoencephalitis. N Engl J Med. 2016;374:1595–6. 10.1056/NEJMc1602964 26958738

[5] Rozé B, Najioullah F, Signate A, Apetse K, Brouste Y, Gourgoudou S, et al.; Neuro-Zika Working Group of Martinique. Zika virus detection in cerebrospinal fluid from two patients with encephalopathy, Martinique, February 2016. Euro Surveill. 2016;21:21. 27123558 10.2807/1560-7917.ES.2016.21.16.30205

[6] Mécharles S, Herrmann C, Poullain P, Tran T-H, Deschamps N, Mathon G, et al. Acute myelitis due to Zika virus infection. Lancet. 2016;387:1481. 10.1016/S0140-6736(16)00644-9 26946926

[7] Plourde AR, Bloch EM. A literature review of Zika virus. Emerg Infect Dis. 2016;22:1185–92. 10.3201/eid2207.151990 27070380 PMC4918175

[8] Zammarchi L, Stella G, Mantella A, Bartolozzi D, Tappe D, Günther S, et al. Zika virus infections imported to Italy: clinical, immunological and virological findings, and public health implications. J Clin Virol. 2015;63:32–5. 10.1016/j.jcv.2014.12.005 25600600

[9] Kutsuna S, Kato Y, Takasaki T, Moi M, Kotaki A, Uemura H, et al. Two cases of Zika fever imported from French Polynesia to Japan, December 2013 to January 2014 [corrected]. Euro Surveill. 2014;19:20683. 10.2807/1560-7917.ES2014.19.4.20683 24507466

[10] Karimi O, Goorhuis A, Schinkel J, Codrington J, Vreden SGS, Vermaat JS, et al. Thrombocytopenia and subcutaneous bleedings in a patient with Zika virus infection. Lancet. 2016;387:939–40. 10.1016/S0140-6736(16)00502-X 26906627

[11] Sarmiento-Ospina A, Vásquez-Serna H, Jimenez-Canizales CE, Villamil-Gómez WE, Rodriguez-Morales AJ. Zika virus associated deaths in Colombia. Lancet Infect Dis. 2016;16:523–4. 10.1016/S1473-3099(16)30006-8 27068488

[12] Zea-Vera AF, Parra B. Zika virus (ZIKV) infection related with immune thrombocytopenic purpura (ITP) exacerbation and antinuclear antibody positivity. Lupus. 2016;•••:0961203316671816. 27694629 10.1177/0961203316671816

[13] Sharp TM, Muñoz-Jordán J, Perez-Padilla J, Bello-Pagán MI, Rivera A, Pastula DM, et al. Zika virus infection associated with severe thrombocytopenia. Clin Infect Dis. 2016;63:1198–201. 27418575 10.1093/cid/ciw476 PMC5176332

[14] Chraïbi S, Najioullah F, Bourdin C, Pegliasco J, Deligny C, Résière D, et al. Two cases of thrombocytopenic purpura at onset of Zika virus infection. J Clin Virol. 2016;83:61–2. 10.1016/j.jcv.2016.08.299 27596376

[15] Williamson DR, Albert M, Heels-Ansdell D, Arnold DM, Lauzier F, Zarychanski R, et al.; PROTECT collaborators; Canadian Critical Care Trials Group; Australian and New Zealand Intensive Care Society Clinical Trials Group. Thrombocytopenia in critically ill patients receiving thromboprophylaxis: frequency, risk factors, and outcomes. Chest. 2013;144:1207–15. 10.1378/chest.13-0121 23788287

